# Collagen type XIV is proportionally lower in the lung tissue of patients with IPF

**DOI:** 10.1038/s41598-023-46733-5

**Published:** 2023-11-08

**Authors:** Mehmet Nizamoglu, Maunick Lefin Koloko Ngassie, Rhode A. Meuleman, Martin Banchero, Theo Borghuis, Wim Timens, Martijn C. Nawijn, Barbro N. Melgert, Irene H. Heijink, Corry-Anke Brandsma, Janette K. Burgess

**Affiliations:** 1 Department of Pathology and Medical Biology, University Medical Center Groningen, University of Groningen, Hanzeplein 1 [HPC EA11], 9713 GZ Groningen, The Netherlands; 2 Groningen Research Institute for Asthma and COPD (GRIAC), University Medical Center Groningen, University of Groningen, Groningen, The Netherlands; 3 Department of Molecular Pharmacology, Groningen Research Institute for Pharmacy, University of Groningen, Groningen, The Netherlands; 4 Department of Pulmonology, University Medical Center Groningen, University of Groningen, Groningen, The Netherlands; 5 W.J. Kolff Institute for Biomedical Engineering and Materials Science-FB41, University Medical Center Groningen, University of Groningen, Groningen, The Netherlands

**Keywords:** Translational research, Respiratory tract diseases

## Abstract

Abnormal deposition of extracellular matrix (ECM) in lung tissue is a characteristic of idiopathic pulmonary fibrosis (IPF). Increased collagen deposition is also accompanied by altered collagen organization. Collagen type XIV, a fibril-associated collagen, supports collagen fibril organization. Its status in IPF has not been described at the protein level yet. In this study, we utilized publicly available datasets for single-cell RNA-sequencing for characterizing collagen type XIV expression at the gene level. For protein level comparison, we applied immunohistochemical staining for collagen type XIV on lung tissue sections from IPF patients and compared it to lung tissue sections from never smoking and ex-smoking donors. Analyzing the relative amounts of collagen type XIV at the whole tissue level, as well as in parenchyma, airway wall and bronchial epithelium, we found consistently lower proportions of collagen type XIV in all lung tissue compartments across IPF samples. Our study suggests proportionally lower collagen type XIV in IPF lung tissues may have implications for the assembly of the ECM fibers potentially contributing to progression of fibrosis.

## Introduction

Idiopathic pulmonary fibrosis (IPF) is the result of aberrant deposition of extracellular matrix (ECM) in the alveolar septa, causing restricted breathing in patients^1^. Currently, the median survival time of patients with IPF is less than for many cancers, and there is no available cure^1^. As the lung ECM structure in IPF is drastically altered and provides a feedback loop that perpetuates disease progression^2,3^, investigating the underlying matrix pathology is expected to improve our understanding of the disease.

Deposition of collagens in the altered ECM in IPF has been well documented, including an increase in collagen types I and III, as well as changes in the organization of collagen fibrils^4,5^. Among other types of collagens, collagen type XIV (COL14) is a fibril-associated collagen with interrupted triple-helices (FACIT)^6^ which consists of two collagenous domains (COL1 and COL2), which are important for the formation of the COL14 triple helical structures and for the binding to other fibrillar collagens, and three non-collagenous (NC) domains (NC1, NC2 and NC3)^7^. Roles for COL14 have been suggested in regulation of fibrillogenesis, cell proliferation and differentiation^6,8,9^. The absence of COL14 in mice resulted in dysregulated biomechanical properties of the skin and altered fibril formation in the tendon^6^. Culture of human skin and mouse 3T3 fibroblasts on immobilized COL14 showed that COL14 inhibits cell proliferation and triggers the differentiation of 3T3 fibroblasts^8^. The presence of COL14 has been demonstrated previously in several tissues including skin, tendon, cornea and lung; especially in the vicinity of the blood vessels, airway smooth muscle cells (ASM) and bronchial epithelium^6,10^. In previous studies by Nishiyama et al.^11^ and Gordon et al.^12^, a role for COL14 in the regulation of mechanical stress has been suggested. We have previously reported the localization of COL14 in regions that would be subjected to high mechanical stress in the lung including the parenchymal region, blood vessels, ASM and bronchial epithelium^10^. Therefore, the presence or absence of COL14 could impact the fibrillogenesis and organization of the collagen type I fibrils and the biomechanical properties of the ECM in different lung compartments and subsequently the lung function.

Unlike abnormal collagen (type I) deposition and organization in IPF which has been described in detail, the role of FACITs in this process or, more specifically, involvement of COL14 in IPF, remains unknown. Previously, in a mouse model of fibrosis, higher levels of COL14 deposition were shown in bleomycin-treated mice compared to control mice^13^. However, the time for development of fibrosis in this model compared to IPF and/or species differences can influence how these results translate to humans. Accordingly, in the current study we aimed to investigate whether there are differences in the COL14 gene expression and protein levels in lung tissue from patients with (end stage) IPF compared to normal human lung tissue.

## Materials and methods

### Data analysis of publicly available single-cell RNA-sequencing datasets

Cell-type specific gene expression of *COL14A1* was analyzed in lung tissue of patients with IPF and non-IPF donors using publicly available single-cell RNA sequencing datasets from the Human Lung Cell Atlas (HCLA) consortium and the IPF atlas dataset from Adams et al.^14,15^. These datasets were analyzed and clustered using HCLA consensus cell-type annotations for 4 major cell groups: endothelial, epithelial, immune and stromal cells for analysis of the expression profile of the Collagen Type XIV Alpha 1 Chain (*COL14A1*) gene which encodes COL14 protein.

### Immunohistochemical staining (IHC)

IHC was used to quantify proteins of interest in lung tissue. Control lung tissues were obtained through resections from patients undergoing surgery for lung cancer, taken from as far away from the tumor region as possible and were macroscopically normal tissue as assessed by pathologists. Fibrotic lungs were obtained from explanted material following transplantations (Table 1). Formalin fixed and paraffin embedded tissue sections of 6 μm thickness were obtained from never-smoker (n = 9), ex-smoker (n = 9) and IPF (n = 12) lungs and were stained for COL14A1 using polyclonal rabbit anti-COL14A1 antibody (HPA023781; Atlas antibodies Inc.) and for COL1A1 using monoclonal mouse, anti-COL1A1 (ab88147; abcam, Waltham, Boston, USA). Secondary polyclonal Goat Anti-Rabbit antibody for detecting COL14A1 (P0488, Dako, Denmark) and secondary polyclonal Rabbit Anti-Mouse antibody for detecting COL1A1 (P0260, Dako) were visualized using NovaRED® Substrate (SK-4800, Vector Laboratories, Canada). Counterstaining was done using hematoxylin. All tissue sections were stained in one batch at the same time to avoid batch effects. Slides were scanned using a Hamamatsu NanoZoomer digital scanner (Hamamatsu Photonic K.K., Shizuoka, Japan) as described previously^10^.

**Table 1:** Patient characteristics for the donors included in this study.

	Never smoker (n = 9)	Ex-smoker (n = 9)	IPF (n = 12)	P value	
				Never smoker vs IPF	Ex-smoker vs IPF
Age (median (min–max))	70 (50–82)	55 (43–62)	58.50 (37–67)	0.0213*	ns*
Sex (M/F)	1/8	3/6	9/3	0.075^#^	ns^#^
FEV1 (pred)^†^ (%, (min–max))	107.5 (80–121)	103.6 (85–127)	49.35 (18–74.50)	0.0002*	0.0002*

*Tested using Mann–Whitney *U* test, ^#^Tested using Fisher’s exact test. ^†^Data was available only n = 8 for all groups. *IPF:* idiopathic pulmonary fibrosis, *FEV1:* forced expiratory volume in 1 s, *ns:* not significant.

The study protocol was consistent with the Research Code of the University Medical Center Groningen (https://umcgresearch.org/w/research-code-umcg) and national ethical and professional guidelines (Code of Conduct for Health Research (coreon.org)). Lung tissues used in this study were derived from leftover lung material after lung surgery and transplant procedures. These materials were not subject to the Medical Research Human Subjects Act in the Netherlands as confirmed by a statement of the Central Ethics Review Board of the University Medical Center Groningen; all biological samples are from archival materials that are exempt from consent in compliance with applicable laws and regulations (Dutch laws: Medical Treatment Agreement Act (WGBO) art 458/GDPR art 9/UAVG art 24). All samples and clinical information were coded before experiments were performed, blinding any identifiable information to the investigators.

### Image analysis

The image analysis was performed for 4 different regions within the scanned images for percentage of positive area as previously described^10^. For whole tissue analysis, scanned images were used after removal of artifacts. For the parenchyma analysis, airways and blood vessels were removed from the whole tissue images. Additionally, specific parts of the airways including the airway wall and the epithelium were examined. An overview of the number of airways and bronchial epithelial images included is presented in Supplementary Table 1. Airways were extracted using Aperio ImageScope software (Leica Biosystems, Amsterdam, The Netherlands). Based on the localization of the staining, different parts of the airways were selected using Adobe Photoshop (Adobe, San Jose, CA, USA). Artifacts and the remaining compartments of the airways which were not used for examination were removed during this step. In the case of IPF airway walls, which have no clear border between the end of the airway wall and the surrounding parenchyma, the distance between the epithelial layer and smooth muscle layer (E-SM distance) was measured. This distance then was multiplied by three to calculate distance from the outside of the smooth muscle layer till the (not visible) parenchyma (SM-P distance). The multiplication by three was calculated based on the ratio between measured E-SM and SM-P distances from IPF airways which were surrounded by still visible parenchyma. The approach is illustrated in Supplementary Fig. 1.

Next, the total tissue area positive for COL14 was quantified by applying color deconvolution to separate the different staining colors^10^. The quantification of the staining was automatically processed using FIJI Image J. Afterwards, the raw data were sorted in RStudio, from which the percentage of area of the tissue present that stained positive for the protein of interest was calculated. The formula used to calculate the percentage of area stained positive for the protein is as follows:

$$Area \, (\%)= \frac{Area\, (Nova\, Red)}{Area\, (Total)} \times 100\%$$

### Statistical analysis

The patient characteristics were evaluated using Mann–Whitney *U* test for age and FEV1 parameters and Fisher’s exact test for sex parameter. Differences between different cell types in the pseudobulk data between non-IPF and IPF groups were tested using a Mann–Whitney *U* test. Differences between never-smoker non-IPF, ex-smoker non-IPF and IPF groups were tested using a Kruskal–Wallis test for whole tissue and parenchyma regions. For airway wall and bronchial epithelium regions, a mixed model analysis was used to test the statistical differences between patients incorporating multiple airway wall and bronchial epithelium images per patient. p < 0.05 was considered significant.

## Results

**Figure 1:**
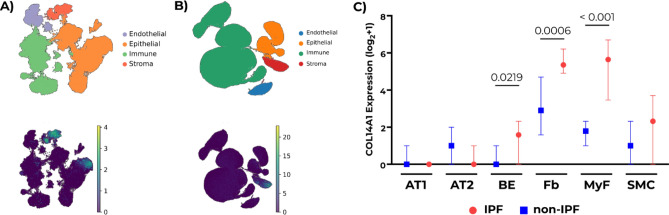
Gene expression profile of COL14A1 in IPF and non-IPF lungs. **A**) COL14A1 expression in healthy lungs (data reconstructed from single-cell RNA-sequencing data obtained from Sikkema et al.^14^. **B**) COL14A1 expression in IPF and non-IPF lungs (reconstructed from single-cell RNA-sequencing data obtained from Adams et al.^15^. **C**) Comparison of COL14A1 counts in IPF and non-IPF lungs across relevant cell types: alveolar type 1 (AT1) and type 2 (AT2), bronchial epithelial cells (BE), fibroblasts (Fb), myofibroblasts (MyF) and smooth muscle cells (SMC). Data are represented as median ± 95% confidence interval. Applied statistical test: Mann–Whitney *U* test. n = 28 for non-IPF, n = 32 for IPF.

We observed *COL14A1* expression in epithelial and stromal cells from control lung tissue in the human lung cell atlas dataset (Fig. 1A), while expression of *COL14A1* in the IPF atlas was mainly restricted to stromal cells (Fig. 1B). Pseudobulk analysis performed on this dataset revealed that *COL14A1* expression in bronchial epithelium, fibroblasts and myofibroblasts of IPF lungs was significantly higher compared to the same cell types in non-IPF lung tissue (Fig. 1C).

**Figure 2:**
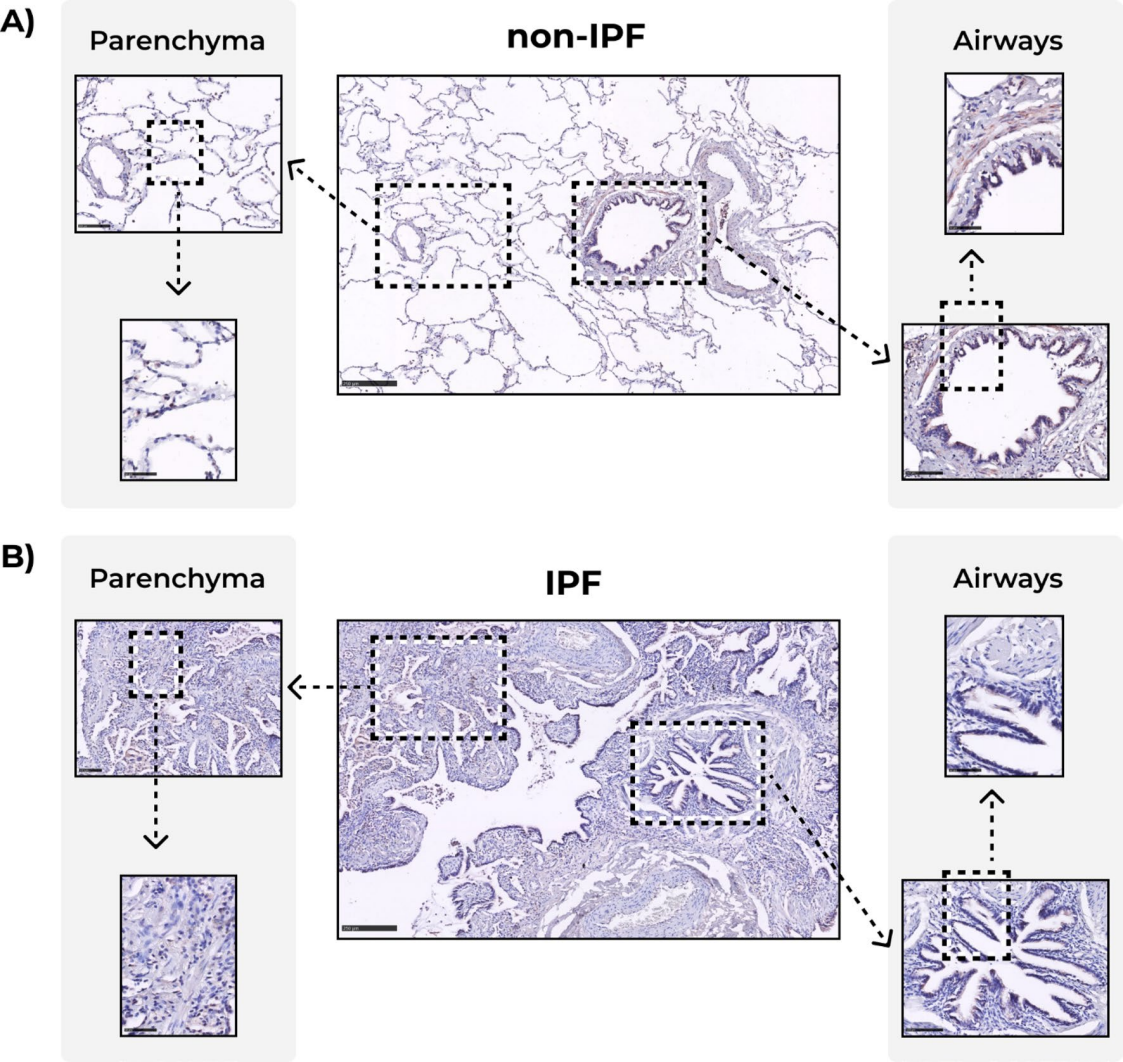
Example images of COL14A1 staining in whole tissue, lung parenchyma, and airways in **A**) non-IPF and **B**) IPF lung samples. Scale bars for intact images: 250 μm, for first level zoom-in: 100 μm and for second level zoom-in: 50 μm. *IPF*: idiopathic pulmonary fibrosis. The contrast and saturation of all images were digitally increased by 5% to enhance visibility.

**Figure 3:**
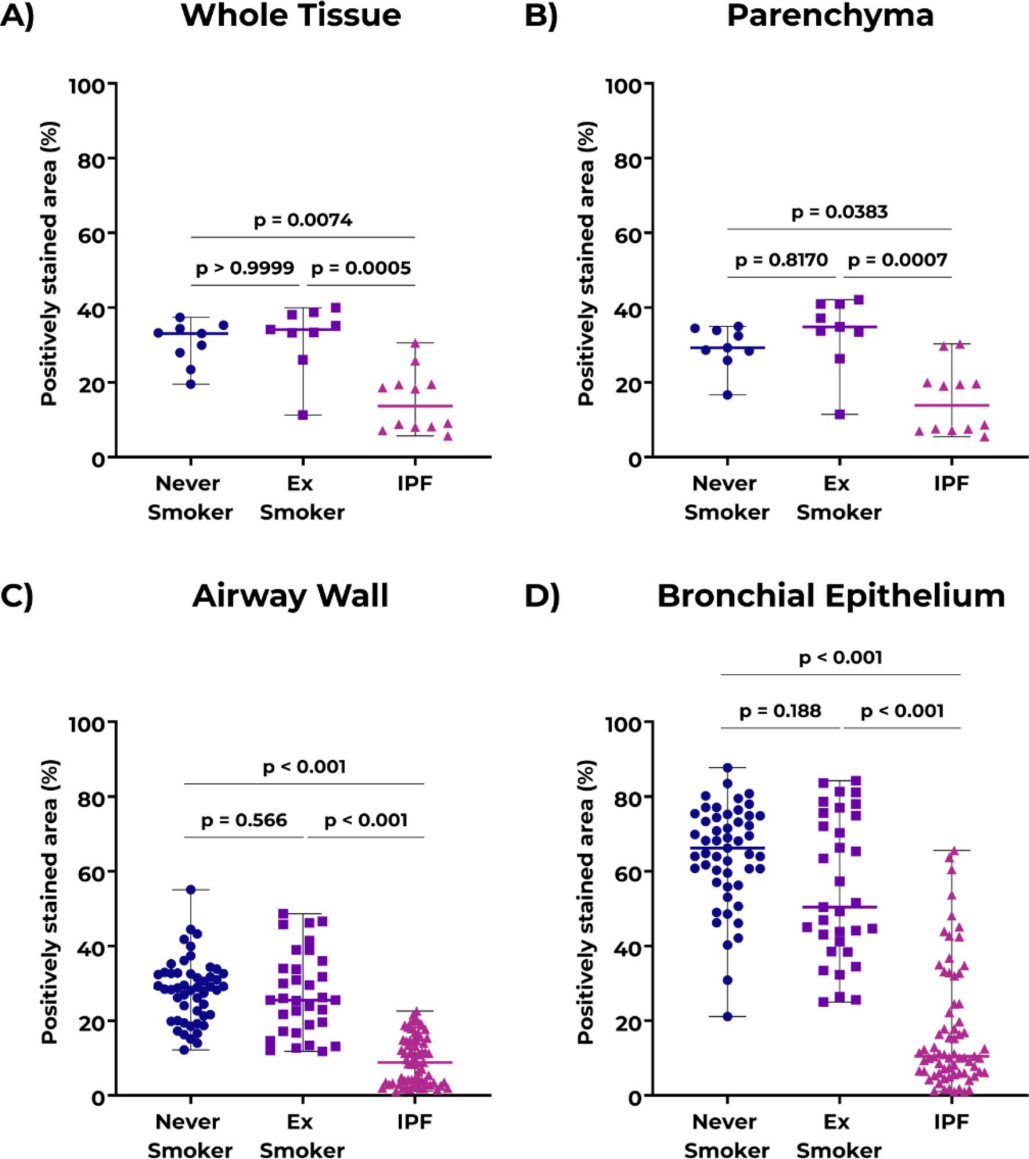
Positively stained area percentage (%) for COL14A1 in: **A**) whole lung tissue, **B**) lung parenchyma, **C**) airway wall, **D**) bronchial epithelium. Data are represented as median ± range. For panels **A**) and **B**), each data point represents individual donors; data were tested using Kruskal–Wallis test with Dunn’s multiple comparisons test for panels. For panels **C**) and **D**), each data point represents individual airway wall or bronchial epithelium (1–9 airway wall or bronchial epithelium images per patient). Data were tested using mixed-model analysis to account for multiple airways per patient. n = 9 for never smoker and ex-smoker groups, n = 12 for IPF. *IPF*: idiopathic pulmonary fibrosis.

**Figure 4:**
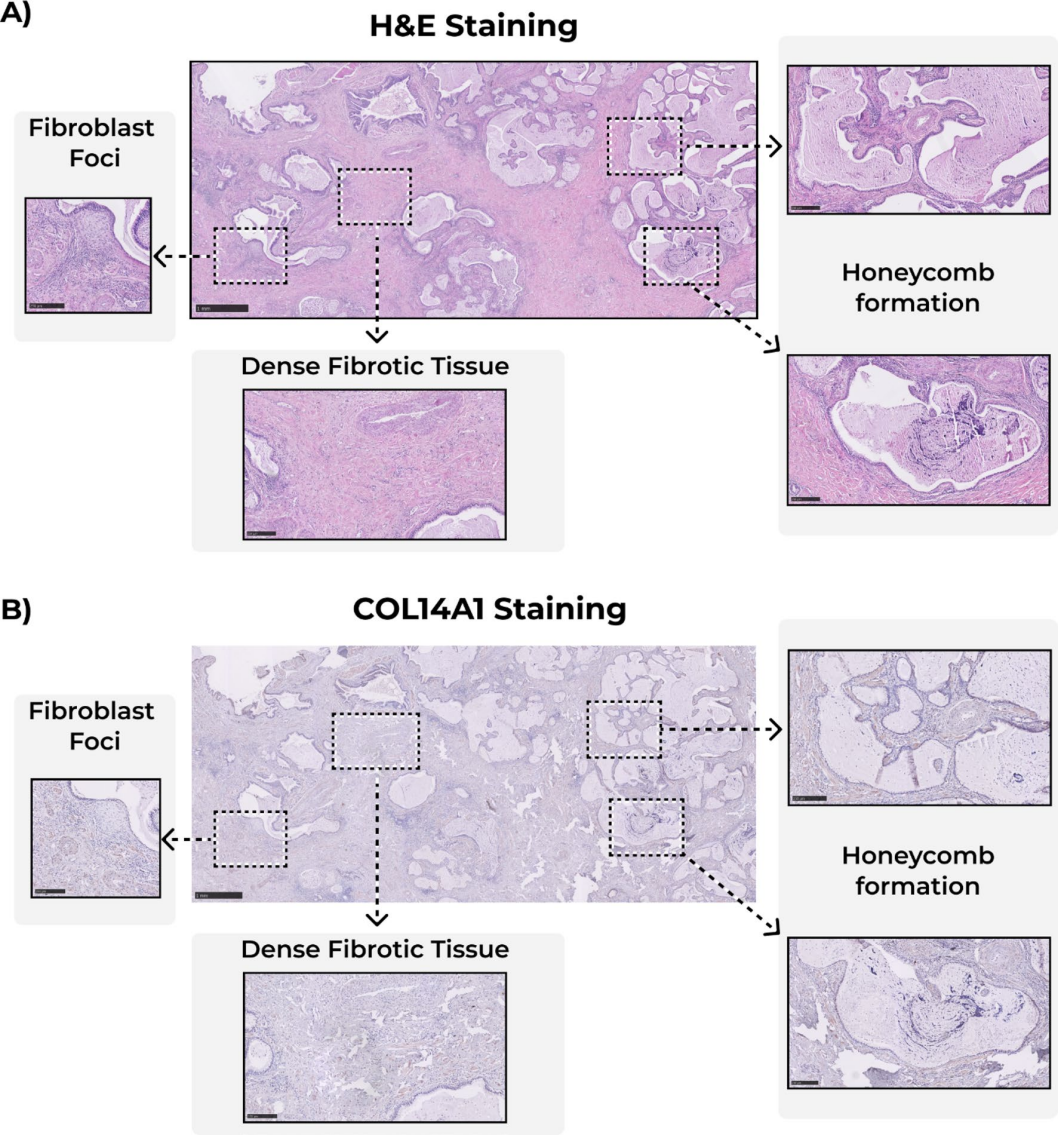
Example images for the fibroblast foci, dense fibrotic tissue and honeycomb formation in **A**) H&E-stained images, and **B**) COL14A1-stained images. Scale bars for intact images: 1 mm, for first level zoom-in: 250 μm.

Immunohistochemical staining of human lung tissue showed that COL14A1 in control tissue was mainly localized in airways and parenchyma (Fig. 2). Image analysis of whole lung tissue sections from IPF and non-IPF donors revealed lower percentages of COL14A1-positive area in tissue from patients with IPF compared to those from both never and ex-smoker controls (Fig. 3A). When focusing on specific regions, we also observed a lower percentage of positively-stained area in lung parenchyma of patients with IPF compared to both non-IPF control groups (Fig. 3B), Lastly, the analysis of airways revealed a lower percentage of positive-stained area of COL14A1 in both airway wall (Fig. 3C) and bronchial epithelium (Fig. 3D) of IPF-derived lung tissue compared to non-IPF controls (both groups). In the whole tissue analysis and analyses of specific tissue compartments, there were no differences in relative positively stained area between never-smoker and ex-smoker non-IPF tissues. In addition to the quantification of the COL14A1 staining on tissue sub-compartments, we have analyzed whether COL14A1 was detected in fibroblast foci, dense fibrotic tissue or honeycomb formation when these structures were present in the sections include in our study (Fig. 4). While moderate amounts of positively-stained areas were visible in dense fibrotic tissue, a lack or relatively weak positive staining in fibroblast foci and honeycomb areas were observed, respectively.

**Figure 5:**
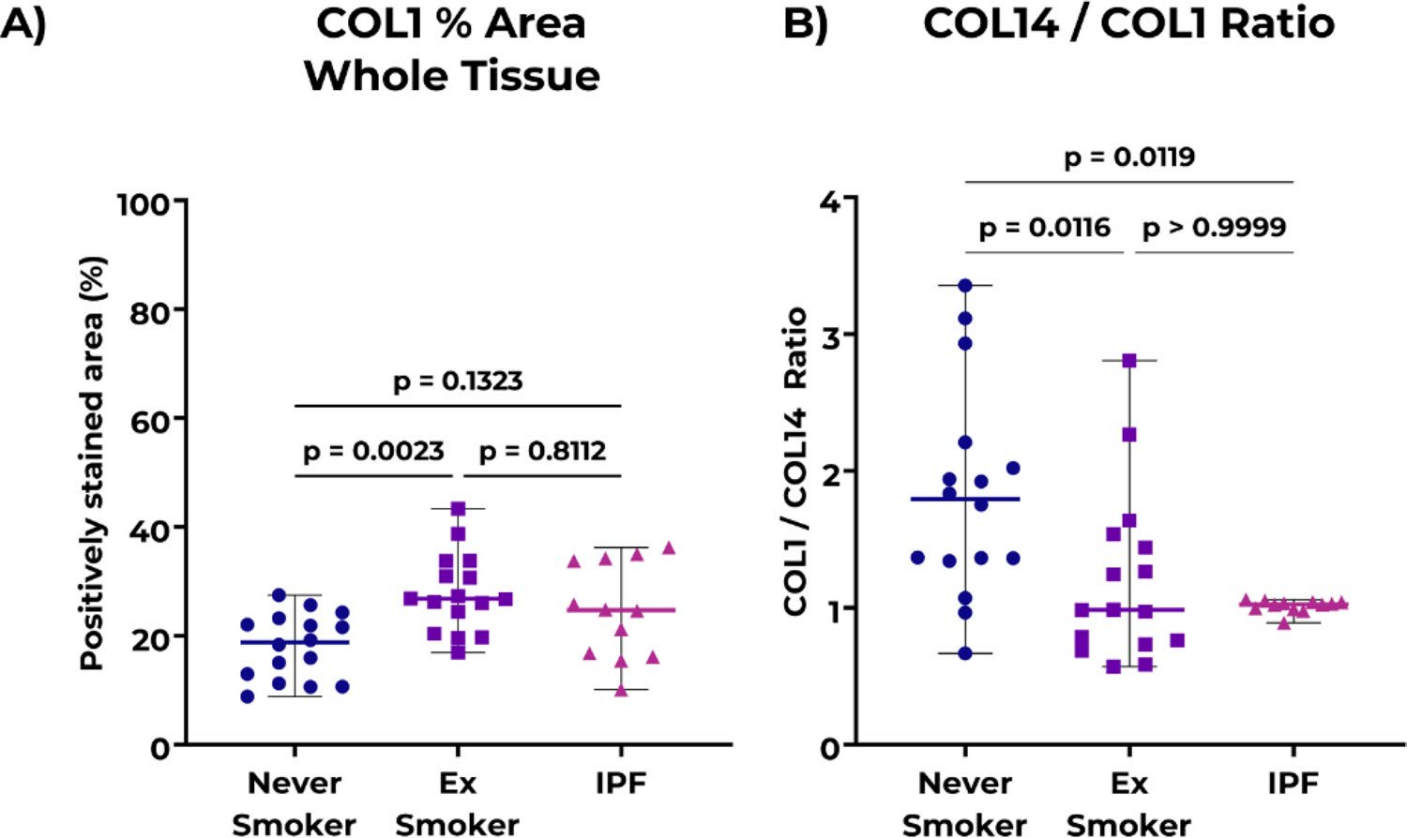
Comparison of positively stained area percentage (%) for COL1 and COL14 in IPF and controls. **A**) Percentage area positively-stained for COL1 in whole tissue lung samples of non-IPF and IPF donors. **B**) Ratio of relative areas for COL14 to COL1 in whole tissue lung samples of non-IPF and IPF donors. Data are represented as median ± range. Each data point represents individual donors; data were tested using Kruskal–Wallis test with Dunn’s multiple comparisons test for panels. n = 16 for never smoker and ex-smoker groups, n = 12 for IPF. *IPF*: idiopathic pulmonary fibrosis.

Lastly, we quantified the percentage area of tissue positively stained for collagen type I (COL1) in the whole tissue of non-IPF and IPF lung samples using the same image analysis pipeline (Fig. 5). Comparing these percentages between groups, we did not observe more relative positively stained area for COLI in lungs of patients with IPF (Fig. 5A) compared to both non-IPF groups. On the other hand, non-IPF ex-smokers had more relative area of COL1 compared to non-IPF never smokers. Subsequently, we compared the ratios of the percentage areas of COL14 to COL1 (COL14/COL1) measured in the lungs of both non-IPF control groups to patients with IPF and found a significantly lower in the COL14/COL1 ratio in patients with IPF compared to the never smoker non-IPF group (Fig. 5B). While the ratio in the ex-smoker group did not differ from the IPF group significantly, it was substantially lower than in the never smoker non-IPF group. When we checked for a possible co-localization of these two proteins, we found a lack intersection of positively stained areas for COL14 in the areas positively stained for COL1 in IPF lung sections (Supplementary Fig. 2), whereas regions of intersection were present in the non-IPF lung sections (Supplementary Fig. 3).

## Discussion

Collectively, our results show proportionally lower COL14 protein expression relative to other ECM components that make up lung tissue in patients with IPF compared to both ex-smoker and never-smoker non-IPF controls. To the best of our knowledge, this is the first report of protein level changes related to spatial location of COL14 in human lung tissue in IPF. In addition, we found a lower ratio of COL14 to COL1 in IPF lungs compared to never smoker non-IPF controls.

In this study, we examined the proportion of lung tissue positive for COL14 in each sample and not the absolute amount of protein. As the total amount of tissue present in IPF lung sections is larger than in controls, it is conceivable there is an increase in absolute amount of COL14, while the proportion of COL14 in comparison to other ECM components is reduced. A previous report of higher mass spectrometry counts of COL14 in IPF lungs compared to non-IPF lungs^16^ suggests that such an absolute increase is likely. However, the ratio of COL14 to COL1 in the same study was lower in IPF lungs compared to control lungs, which is consistent with our findings. While an early study analyzing the amount of COL14 in IPF lungs semi-quantitatively reported higher COL14 in established fibrotic areas and lower COL14 in fibroblastic foci^17^, these results portray an absolute amount of protein rather than relative to other ECM components as depicted in our study.

The single-cell analyses using the IPF atlas^9^ to investigate the changes in *COL14A1* expression in lung tissue from patients with IPF compared to non-IPF controls have revealed a higher expression in fibroblasts and myofibroblasts, as well as in basal cells isolated from IPF lungs. These differences in *COL14A1* mRNA expression between patients with IPF and controls are in the opposite direction than the differences observed at the protein level in our IHC stainings. It has been shown previously that correlations between the steady-state number of protein molecules per cell and their associated mRNA molecules are weak^18^. Proportional changes between the relative abundances of the proteins and the relative abundances of the mRNA of these proteins is also not guaranteed^19^, and possible mechanisms for this lack of proportion between mRNA and protein levels have been discussed elsewhere^20^. In addition, the status of the proteins already deposited in the ECM is unlikely to be determined by their mRNA. It might be possible that the increased gene expression of *COL14A1* reflects an attempt of the structural cells of the lung to compensate for the reduced COL14 protein levels observed in lung tissue.

Regarding our results from the COL1 image analysis, we did not observe a relative higher amount of the percentage positively stained areas in IPF lung samples compared to control groups. This lack of increase, however, does not mean a lack of absolute increase as more deposition of COL1 in IPF lungs has been previously established^21^. Even with this, recent proteomics data from Hoffman et al. show a lack of absolute increase in COL1A1 in decellularized IPF lung samples^22^. An interesting observation from our study was the influence of smoking on the lung ECM proteins in the lungs of non-IPF donors. While relative amounts of COL14 did not differ based on the smoking status of the non-IPF controls, the percentage positively stained areas for COL1 differed significantly between the never smoker and ex-smoker groups. Considering that the lungs of ex-smoker donors did not have visible fibrotic regions, it is possible that this higher relative amount of COL1 reflects more absolute amounts. In IPF lung tissue, the samples had COL14 positive areas in dense fibrotic tissue while the fibroblastic foci and honeycomb regions were mostly devoid of COL14. Further research is needed to investigate how the collagen organization differs in these dense fibrotic tissue areas with respect to the COL14 presence as well as relating these changes to lack of COL14 in fibroblastic foci or honeycomb regions.

How COL14 is involved in the regulation of human lung ECM structure and how changes in its proportional expression influence fibrotic responses in IPF is currently unknown. As FACITs regulate the structural organization of fibrillar collagens, it can be argued that lower proportions of COL14 in IPF lungs contribute to the reported imbalance between organized and disorganized fibrillar collagen in IPF^5^. More research correlating fibrillar collagen organization with FACITs such as COL14 will increase understanding of the involvement of COL14 in the development and/or progression of fibrotic responses. A recent study using mice deficient in *COL14A1* found increased stiffness and abnormal collagen fibril organization in the corneas of *COL14A1-*knockout mice compared to wildtypes^23^. These observations parallel well-documented increases in the stiffness of lungs from patients with IPF^16^. Together with our observations reported here, it is tempting to speculate that COL14 protein levels in IPF lungs do not mirror the increase in other ECM proteins in lung tissue during fibrosis, thereby leading to more disorganized collagen fibers with higher stiffness in lungs of patients with IPF.

### Supplementary Information

Supplementary Information.

## Data Availability

Single-cell RNA-sequencing data used to reach the conclusions are publicly available through the following IDs: EGAS00001004344 for Human Lung Cell Atlas data (link: https://cellxgene.cziscience.com/e/066943a2-fdac-4b29-b348-40cede398e4e.cxg/) and GSE135893 for IPF Cell Atlas data. The remaining data can be made available upon reasonable request from the corresponding author (Prof. Janette Burgess).
