# Supplementary material for: Collagen type XIV is proportionally lower in the lung tissue of patients with IPF
Source: Sci Rep. 2023 Nov 8;13:19393. doi: 10.1038/s41598-023-46733-5 (PMC10632429; doi:10.1038/s41598-023-46733-5)
Supplement: Supplementary file 1 — Supplementary Information. [file 41598_2023_46733_MOESM1_ESM.docx]

**
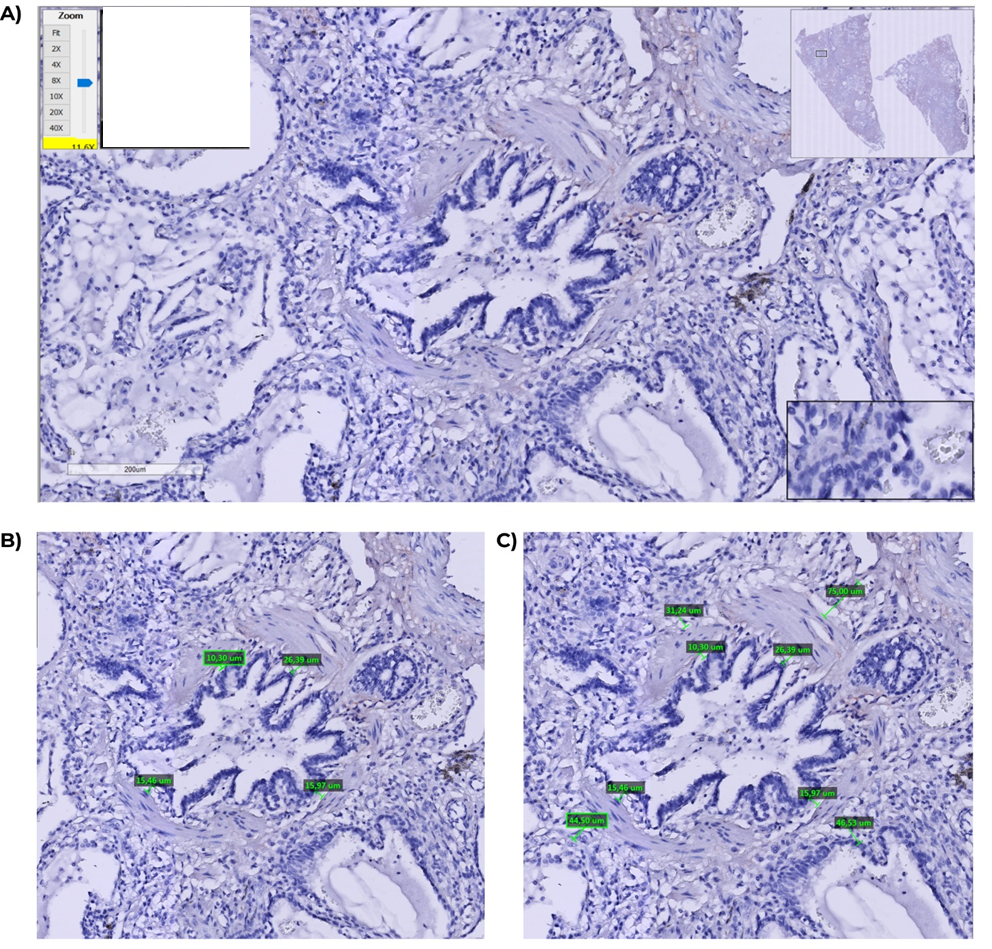
Supplementary Figure 1:** Example of how to determine smooth muscle-parencyhma distances of a fibrotic airway. (A) Measurements were performed on the airway in the Aperio ImageScope program. (B) The airway with measured epithelial layer and smooth muscle layer distances. (C) The airway with both measured epithelial layer and smooth muscle layer distances and calculated smooth muscle-parencyhma distances. The smooth muscle-parencyhma distances are measured starting from the outside of the smooth muscle layer.

**Supplementary Table 1:** Number of airway and bronchial epithelium images analyzed per patient from ex-smoker, never smoker and IPF groups.

| **Group** | **Donor ID** | **Number of Airway Layers** | **Number of Bronchial Epithelial Layers** |
| --- | --- | --- | --- |
| **Ex-smoker** | Donor 1 | 5 | 5 |
| **Ex-smoker** | Donor 2 | 1 | 1 |
| **Ex-smoker** | Donor 3 | 2 | 2 |
| **Ex-smoker** | Donor 4 | 4 | 4 |
| **Ex-smoker** | Donor 5 | 2 | 2 |
| **Ex-smoker** | Donor 6 | 6 | 6 |
| **Ex-smoker** | Donor 7 | 2 | 2 |
| **Ex-smoker** | Donor 8 | 5 | 5 |
| **Ex-smoker** | Donor 9 | 6 | 6 |
| **Never-smoker** | Donor 1 | 5 | 5 |
| **Never-smoker** | Donor 2 | 9 | 9 |
| **Never-smoker** | Donor 3 | 2 | 2 |
| **Never-smoker** | Donor 4 | 8 | 8 |
| **Never-smoker** | Donor 5 | 5 | 5 |
| **Never-smoker** | Donor 6 | 9 | 9 |
| **Never-smoker** | Donor 7 | 1 | 1 |
| **Never-smoker** | Donor 8 | 4 | 4 |
| **Never-smoker** | Donor 9 | 7 | 7 |
| **IPF** | Donor 1 | 5 | 5 |
| **IPF** | Donor 2 | 9 | 9 |
| **IPF** | Donor 3 | 8 | 8 |
| **IPF** | Donor 4 | 5 | 5 |
| **IPF** | Donor 5 | 8 | 8 |
| **IPF** | Donor 6 | 9 | 9 |
| **IPF** | Donor 7 | 7 | 7 |
| **IPF** | Donor 8 | 4 | 4 |
| **IPF** | Donor 9 | 3 | 3 |
| **IPF** | Donor 10 | 5 | 5 |
| **IPF** | Donor 11 | 2 | 2 |
| **IPF** | Donor 12 | 4 | 4 |


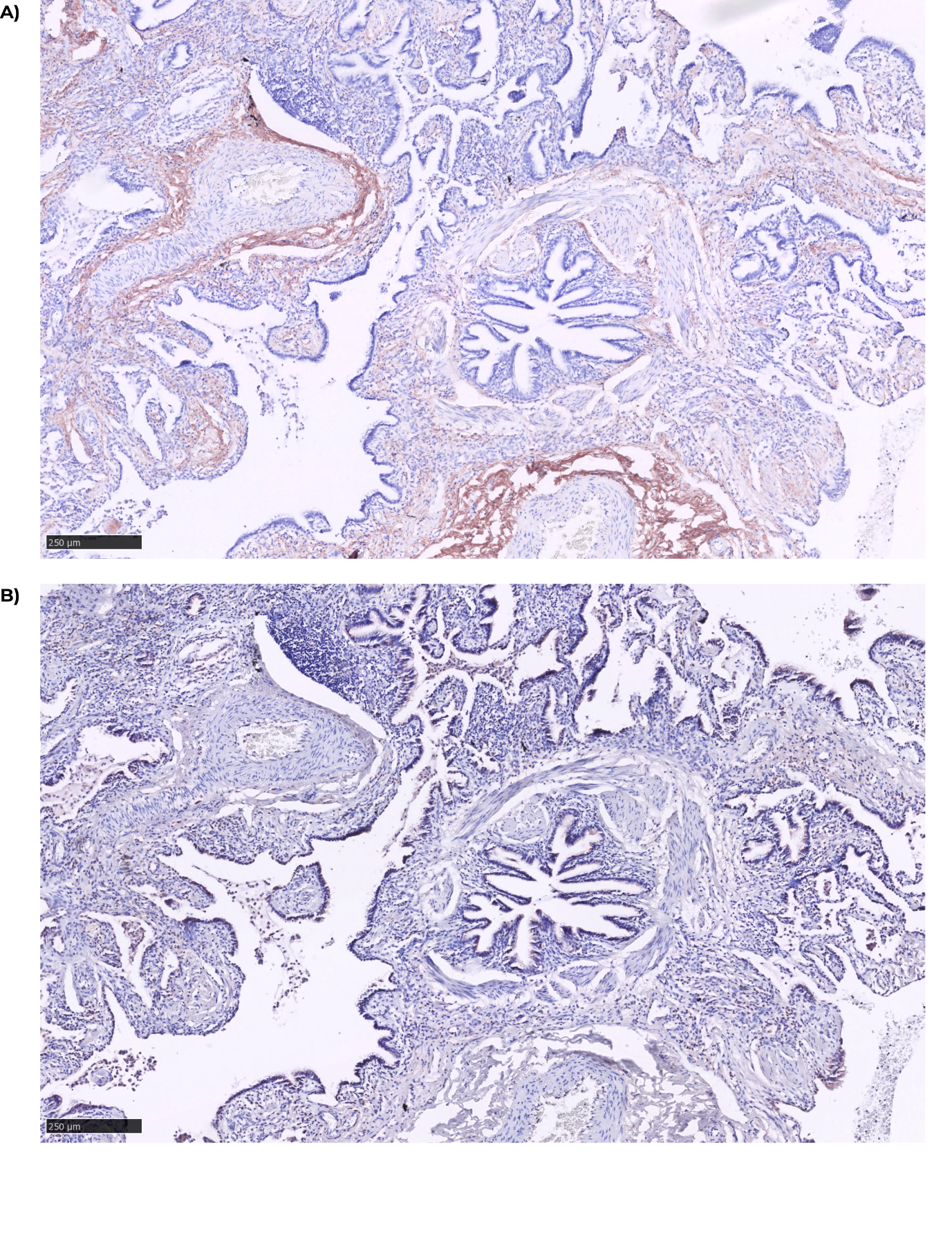


**SM**

**AA**

**VA**

**BE**

**ASM**

**VSM**

**Supplementary Figure 2:** Example images of immunohistochemical staining performed on sections of IPF lung tissue against A) COL1A1 and B) COL14A1 in whole tissue. Scale bars: 250 μm. The contrast and saturation of all images were digitally increased by 5% to enhance visibility. AA: airway adventitia, BE: bronchial epithelium, ASM: airway smooth muscle, VSM: vessel smooth muscle, VA: vessel adventitia, SM: submucosa


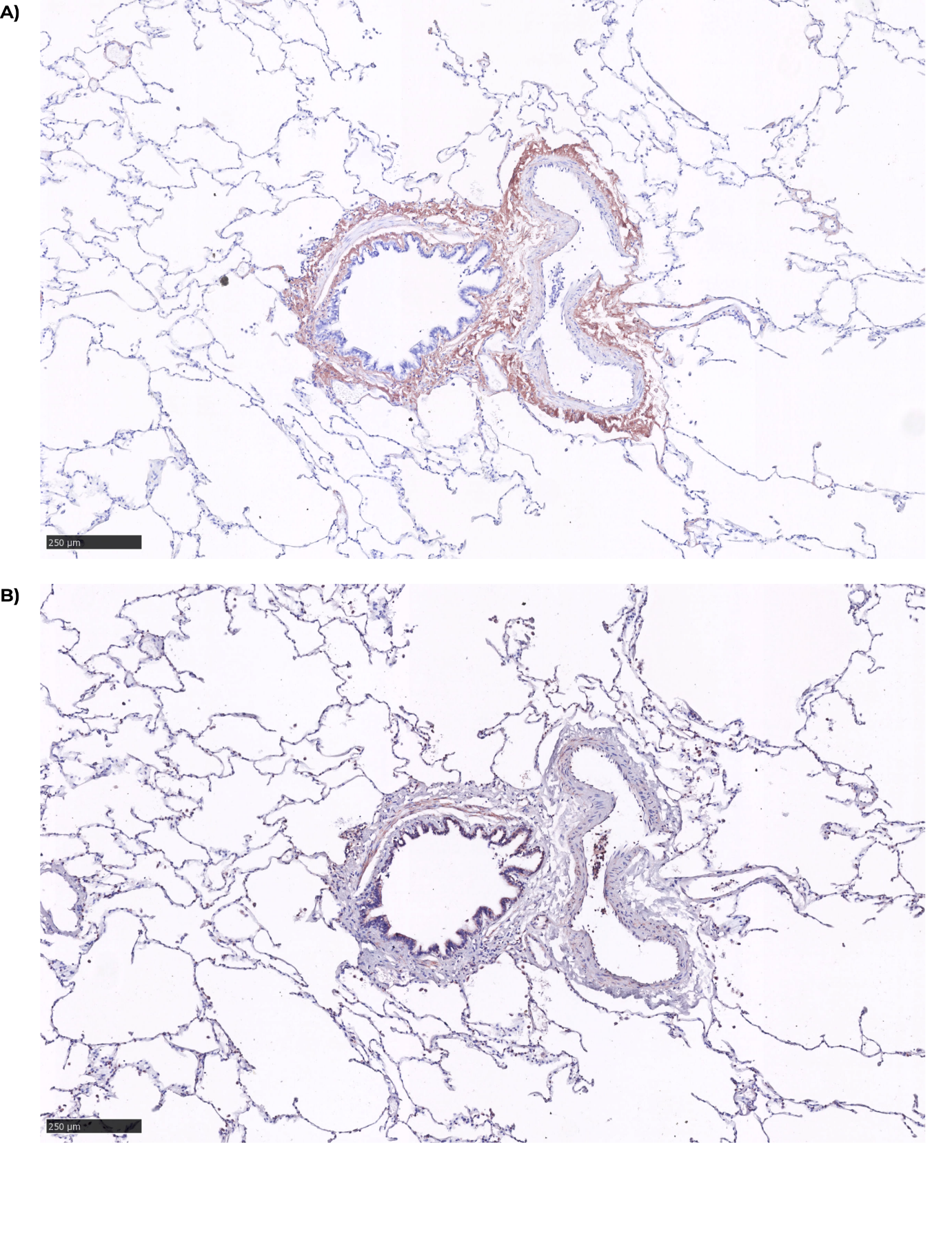


**BE**

**ASM**

**P**

**P**

**AA**

**SM**

**VA**

**VSM**

**Supplementary Figure 3:** Example images of immunohistochemical staining performed on sections of non-IPF lung tissue against A) COL1A1 and B) COL14A1 in whole tissue. Scale bars: 250 μm. The contrast and saturation of all images were digitally increased by 5% to enhance visibility. AA: airway adventitia, BE: bronchial epithelium, ASM: airway smooth muscle, VSM: vessel smooth muscle, P: parenchyma, VA: vessel adventitia, SM: submucosa
